# The Structure of a Plant Tyrosinase from Walnut Leaves Reveals the Importance of “Substrate-Guiding Residues” for Enzymatic Specificity

**DOI:** 10.1002/anie.201506994

**Published:** 2015-10-16

**Authors:** Aleksandar Bijelic, Matthias Pretzler, Christian Molitor, Florime Zekiri, Annette Rompel

**Affiliations:** Institut für Biophysikalische Chemie, Fakultät für Chemie Universität Wien, Althanstraße 14, 1090 Wien (Austria) http://www.bpc.univie.ac.at

**Keywords:** enzyme catalysis, metalloenzymes, oxidoreductases, structure elucidation, tyrosinase

## Abstract

Tyrosinases and catechol oxidases are members of the class of type III copper enzymes. While tyrosinases accept both mono- and *o*-diphenols as substrates, only the latter substrate is converted by catechol oxidases. Researchers have been working for decades to elucidate the monophenolase/diphenolase specificity on a structural level and have introduced an early hypothesis that states that the reason for the lack of monophenolase activity in catechol oxidases may be its structurally restricted active site. However, recent structural and biochemical studies of this enzyme class have raised doubts about this theory. Herein, the first crystal structure of a plant tyrosinase (from *Juglans regia*) is presented. The structure reveals that the distinction between mono- and diphenolase activity does not depend on the degree of restriction of the active site, and thus a more important role for amino acid residues located at the entrance to and in the second shell of the active site is proposed.

Tyrosinases are type III copper-containing oxidoreductases that are found in a wide range of organisms distributed over all domains of life.^1^ Tyrosinase catalyzes the reactions that provide the starting material for melanin biosynthesis, namely the *ortho*-hydroxylation of monophenols to *o*-diphenols (monophenolase activity, EC 1.14.18.1) and the subsequent oxidation of *o*-diphenols to the corresponding *o*-quinones (diphenolase activity, EC 1.10.3.1), which are both coupled to the reduction of molecular oxygen to water.^2^ During the catalytic cycle, the dinuclear copper center passes through three different oxidation states. In the resting *met* form, the copper atoms (Cu^II^) are bridged by a hydroxide ion or water molecule. The *deoxy* form represents the reduced (Cu^I^) state, which is converted into the reactive *oxy* form upon oxygen binding.^3^ Silencing of walnut tyrosinase (*jr*TYR) induces a lesion mimic phenotype in walnut leaves, presumably owing to tyramine-mediated cell death.^4^

In the past decades, the catalytic mechanisms of both tyrosinases and catechol oxidases have been intensively investigated by X-ray crystallography, among other techniques. X-ray structure analysis has revealed high similarity within the active site of the two enzyme types, with only slight differences, most prominently a bulky amino acid that limits substrate access to the active site, which was first identified in the pioneering crystal structure of the catechol oxidase from *Ipomoea batatas*.^5^ This residue was considered crucial for controlling mono-/diphenolase specificity and therefore the term “blocker residue” was coined. The theory was further supported by the first tyrosinase crystal structure from *Streptomyces castaneoglobisporus*, the blocker position of which is occupied by glycine.^6^ However, the structure–function relationship is still a matter of debate.

Herein, the first high-resolution crystal structure of a plant tyrosinase in its active form is presented. This tyrosinase was purified from walnut leaves and has both monophenolase activity and a bulky residue at the blocker position.^7^, ^8^

*jr*TYR was isolated from walnut leaves^7^ and the structure was determined by X-ray crystallography to a resolution of 1.8 Å (PDB ID: 5CE9). The core structure of *jr*TYR is almost identical to reported polyphenol oxidase (PPO) structures.^6^, ^9^–^12^ It shares the highest structural similarity with its plant relatives, the catechol oxidases from *Ipomoea batatas* (*ib*CO, sequence identity of the main core 56.6 %)^5^ and *Vitis vinifera* (*vv*CO, 63.7 %).^13^ The active-site region containing the dinuclear copper center is formed by a bundle of four α-helices (α4, α5, α12, and α14; Figure 1 A). Each active-site copper ion is coordinated by three histidine residues (His; Figure 1 B). Copper A (CuA) is coordinated by His87, His108, and His117, where His87 and His117 are located on α-helices (α4 and α5) and His108 on a loop. The Cε atom of His108 forms a thioether bond with the sulfur atom of an adjacent cysteine (Cys91), thereby resulting in limited flexibility of His108. Copper B (CuB) is coordinated by His239, His243, and His273, which are all located on α-helices.

**Figure 1:**
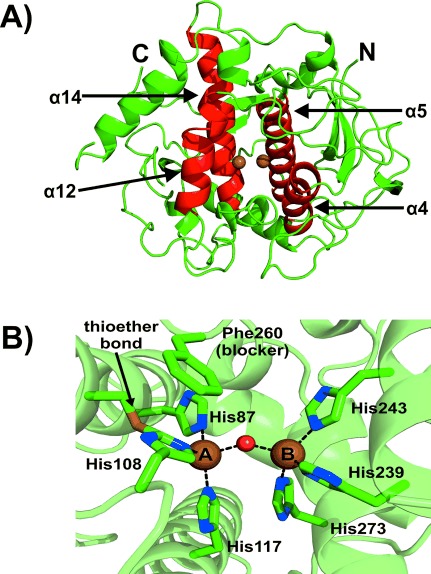
Overall and active-site structure of *jr*TYR. A) The overall structure is shown as green cartoon with the four α-helical bundles forming the active site colored in red. B) The active site with the copper-coordinating histidine residues, blocker residue, and thioether bond are illustrated as stick models (green C, blue N, yellow S). The rest of the protein is shown as a green cartoon with 50 % transparency. The copper atoms are shown as brown spheres with the bridging solvent as a small red sphere.

*jr*TYR was crystallized in its resting *met* form with a Cu–Cu distance of 4.0 Å, which is similar to the *met* form of *vv*CO (4.2 Å).^13^ The two copper centers are bridged by a solvent molecule, most probably a hydroxide anion, which is 2.1 Å from each copper ion. With all coordinating histidines and the solvent molecule, CuA exhibits an intermediate geometry between a distorted tetrahedron and a trigonal bipyramid with one unoccupied coordination site, whereas CuB exhibits an almost perfect tetrahedral geometry with the solvent molecule in apical position (Figure 1 B).

The structure contains two disulfide bonds (Cys11–Cys26 and Cys25–Cys88), which on the one hand stabilize N-terminal loops by anchoring them to the main core, and on the other hand are associated with copper incorporation, since *jr*TYR contains the well-conserved tyrosinase CXXC motif (C88 A-Y-C91), which has been reported to be crucial for copper uptake.^11^ The two disulfide bonds in *jr*TYR are located next to each other, at a distance of about 8 Å. They represent the shortest path from outside the enzyme into the active site (about 16 Å) and thus could thus play a similar role to the CXXC motif in other tyrosinases or copper chaperones (Figure S1 in the Supporting Information).^14^

At the blocker residue position above CuA, *jr*TYR contains a phenylalanine residue (Phe260; Figure 1 B). This is interesting because the presence of a bulky blocker residue has been associated with catechol oxidases.^5^, ^6^ It has been postulated that the bulky phenylalanine in catechol oxidases prevents the binding of substrates to CuA, thereby resulting in the lack of monophenolase activity.^9^, ^15^, ^16^ This led to the assumption that monophenols bind to CuA and diphenols to CuB.^17^ However, Goldfeder et al. replaced the blocker-position Val218 in tyrosinase from *Bacillus megaterium* (*bm*TYR) with a bulky phenylalanine in order to hamper monophenolase activity (mimicking a catechol oxidase), but the monophenolase activity surprisingly increased and the diphenolase activity decreased.^18^ The same group provided crystal structures (*bm*TYR soaked with different substrates) that revealed that both monophenols and diphenols bind to CuA in *bm*TYR, and therefore they concluded that both substrate types also bind to CuA in catechol oxidases.^19^ It was suggested that the limited flexibility of the CuA site (thioether bond) in combination with a bulky blocker residue is responsible for the absence of monophenolase activity in catechol oxidases, since both restrictions would prevent substrate rotation, which is believed to be necessary during the hydroxylation of monophenols.^17^, ^19^ However, monophenolase activity has been unambiguously proven for *jr*TYR, even though the herein presented crystal structure shows both a bulky blocker residue above CuA and the rigidifying thioether bond.^7^ This clearly demonstrates that a restricted active site cannot be the reason for mono-/diphenolase specificity.

These findings were further supported by a comparison of the *jr*TYR structure with those of the plant catechol oxidases *ib*CO (PDB ID: 1BT3)^5^ and *vv*CO (PDB ID: 2P3X).^13^ Although possessing different functionalities, the three structures are nearly identical, with no difference in their amino acid sequence within a radius of 5.5 Å from each copper center (Figure 2). Only in the second shell of the active site (here defined as amino acid residues located at least 6 Å away from each copper atom) do the structures become slightly different, with the clearest difference in the positioning of the blocker residue (Figure 2). Phe261 in *ib*CO covers CuA totally and thus seems to justify the term “blocker”, however another structure of *ib*CO with the bound inhibitor phenylthiourea (PTU; PDB ID: 1BUG)^5^ demonstrated flexibility of the Phe261 side chain, which was shifted away from CuA upon PTU binding. The same was reported for *vv*CO, where the crystal structure of the free enzyme shows partial shielding of CuA by Phe260 (Figure 2, inset) but a molecular dynamic simulation study revealed phenylalanine flexibility that allows substrate access to CuA.^20^ In the case of *jr*TYR, CuA is already freely accessible in the resting state (Figure 2, inset). It is not clear whether this is the result of Phe260 rotation or due to a fixed position, because every analyzed data set (in total five) showed the same position for the side chain of Phe260. It can be concluded that at least for plant polyphenol oxidases, the term “blocker” for the residue above CuA is misleading and should be reconsidered.

**Figure 2:**
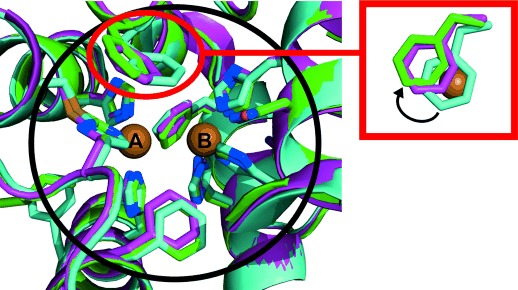
Superimposition of the active sites of *jr*TYR, *ib*CO, and *vv*CO. Proteins are shown in cartoon representation with the active-site residues illustrated as stick models and each structure colored differently: *jr*TYR in green, *ib*CO in cyan, and *vv*CO in purple (green/cyan/purple C, blue N, yellow S, red O). The big black circle delineates the area of the first shell of the active site (ca. 5.5 Å around each copper). The red circle with the associated inset indicates the different positions of the phenylalanine residues above CuA as seen from the top, with CuA (from *jr*TYR) drawn as a brown sphere. The phenylalanine of *ib*CO (cyan) covers CuA totally, whereas only partial CuA shielding can be observed for the phenylalanine of *vv*CO (purple). The position of the phenylalanine side chain of *jr*TYR (green) leads to no shielding. The black arrow in the inset indicates the shift from total CuA coverage (*ib*CO, cyan) to no coverage (*jr*TYR, green).

Second-shell residues at the active-site entrance are thus crucial for the difference in activity, even though there are only few differences in residues between the plant PPOs. However, some of these differences lead to distinct electrostatic behavior at the respective positions, for example, the residue located above the second CuB-coordinating His (His243 in *jr*TYR). *jr*TYR contains a small hydrophobic leucine residue (Leu244) at this position and in contrast, *ib*CO and *vv*CO possess long and positively charged arginine (Arg245) and lysine (Lys244) residues, respectively, both of which are able to stabilize acidic functionalities of substrates. This suggests that electrostatic interactions are important during substrate binding to both enzyme types and could affect tyrosinase specificity.^21^

In one chain of the crystallographic dimer of *jr*TYR, an interesting difference electron density in the active site was found, which looks like the blurred density of a bound ligand (Figure 3 A). This density seems to indicate a pathway from the protein surface into the active site and most probably originates from influxing solvent molecules. One part of this density overlaps with CuA and it can be implied that incoming substrates could follow this pathway and thus be directed towards CuA, which was also reported in the substrate-bound structures of *bm*TYR^19^ [PDB IDs: 4P6R (bound *l*-tyrosine), 4P6S (bound *l*-DOPA) and 4P6T (bound *p*-tyrosol)]. Superimposition of *jr*TYR with the substrate-bound structures of *bm*TYR overlapped very well (root mean square deviation (rmsd)^Cα^ of ca. 1.3 Å, 647 matched atoms) and revealed that the substrate orientation was compatible with the density found in the active site of *jr*TYR, thus supporting this pathway (Figure 3 B). Kinetic studies were performed with the same substrates to support the superimposition analysis and the results show that all of these phenolic compounds are readily accepted as substrates, with significantly faster turnover for the monophenols lacking a carboxylate moiety (Figure S2 and Table S2 in the Supporting Information). This could be due to the aforementioned leucine residue (Leu244), which preferentially stabilizes more-hydrophobic substrate moieties. In contrast, the *bm*TYR structure contains an arginine residue (Arg209) at this position and thus is able to form hydrogen bonds with substrates possessing a carboxylic group, as shown in the tyrosine- and l-DOPA-bound structures.^19^

**Figure 3:**
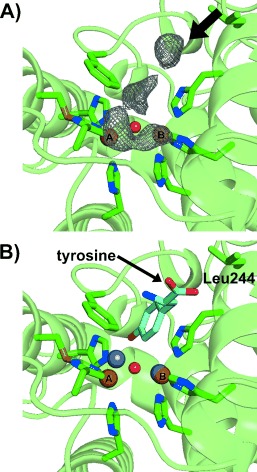
Pathway into the active site. A) The mFo-DFc map (contoured at 3.0 σ) of the electron density marking the pathway into the active site is illustrated as a gray mesh. The arrow indicates the direction of the pathway. The six copper-coordinating histidine residues, the phenylalanine residue above CuA, the second-shell Leu244, and the thioether bridge are shown as stick models (green C, blue N, yellow S), whereas the remaining structure is represented as a green cartoon with 50 % transparency. The two copper ions are illustrated as brown spheres, with the bridging solvent molecule as a small red sphere. B) Superimposition of tyrosine from the *bm*TYR+tyrosine structure (PDB ID: 4P6R; shown as a stick model with cyan C, blue N, red O) with *jr*TYR (represented as in (A)). The copper ions from *jr*TYR are shown as brown spheres and the superimposed zinc ions from *bm*TYR+tyrosinase are shown as silver spheres. The superimposed substrate is indicated to exhibit the same orientation as the path in Figure 3 A. Leu244 is located within the second shell of the active site and it is in close proximity to the carboxylic tail of tyrosine. Substrates possessing a hydrophobic tail instead of a carboxylic moiety are able to interact (hydrophobically) with Leu244 and are thus stabilized (as indicated by the kinetic data).

Furthermore, the superimposition revealed that the bulky residue Phe260 in *jr*TYR is orientated in such a way that it is able to exhibit at least weak T-shaped π–π interaction with the aromatic ring of a substrate, thus suggesting a new role for Phe260, which, together with the CuB-coordinating His243, seems to build a specifically formed gate leading into the active site that can only be passed by substrates in the correct orientation (Figure S3 in the Supporting Information). The aromatic rings of all of the superimposed substrates were located between Phe260 and His243 and are stabilized by the parallel His243 through cation–π interactions, which has also been shown in *bm*TYR.^19^ Recently, it was reported that mutation of the Phe273 above CuA into alanine in *cg*AUS1 (a plant PPO involved in aurone biosynthesis) results in significant loss of diphenolase activity, which strongly supports the proposed importance of this residue for substrate orientation and binding at the active site.^22^

A binding mechanism is proposed in which the substrate is pre-orientated at the active-site entrance by second-shell residues through electrostatic and hydrophobic interactions. At the same time, the substrate is deprotonated by a well-conserved water molecule, which is stabilized by Glu235 and Asn240 (Figure S4 in the Supporting Information).^1^, ^19^ Afterwards it is finally orientated by His243 and Phe260 and approaches CuA in a slightly shifted manner so that the *o*-position of the substrate phenol ring is directed to the copper-bridging oxo ligands (assuming enzyme being in the *oxy* form). The hydroxylation reaction could then be assisted by further solvent molecules serving as proton donors and acceptors to facilitate the reaction. Subsequently, the diphenolic intermediate undergoes oxidation to give the final *o*-quinone. This “substrate-guiding residues” mediated mechanism does not need much substrate rotation and needs no rearrangement of the active site, since the substrate is already introduced in the correct orientation for both reactions.

In summary, the first plant tyrosinase structure has been presented and reveals that the presence of a bulky residue above CuA and flexibility around the CuA site are not responsible for the lack of monophenolase activity in plant PPOs, as assumed previously. These findings are in accordance with recent developments in this field, which shed doubts upon the “classic” role of the bulky residue. Therefore, it is suggested that the electrostatic environment, conformation, and type of second-shell residues at the active-site entrance could be the key for mono-/diphenolase specificity.

## Experimental Section

The active enzyme was isolated, purified and subsequently crystallized as described previously.^7^, ^23^ Descriptions of the methods, the data collection statistics (Table S1) and relevant references for the structure elucidation by X-ray crystallography may be found in the Supporting Information.

## Supporting Information

As a service to our authors and readers, this journal provides supporting information supplied by the authors. Such materials are peer reviewed and may be re-organized for online delivery, but are not copy-edited or typeset. Technical support issues arising from supporting information (other than missing files) should be addressed to the authors.

miscellaneous_information
