# Supplementary material for: The Structure of a Plant Tyrosinase from Walnut Leaves Reveals the Importance of “Substrate-Guiding Residues” for Enzymatic Specificity
Source: Angew Chem Int Ed Engl. 2015 Oct 16;54(49):14677–80. doi: 10.1002/anie.201506994 (PMC4678486; doi:10.1002/anie.201506994)
Supplement: Supplementary file 1 [file anie0054-14677-sd1.pdf]

Angewandte Chemie
Eine Zeitschrift der Gesellschaft Deutscher Chemiker

Supporting Information

The Structure of a Plant Tyrosinase from Walnut Leaves Reveals the Importance of "Substrate-Guiding Residues" for Enzymatic Specificity

*Aleksandar Bijelic, Matthias Pretzler, Christian Molitor, Florime Zekiri, and Annette Rompel**

anie_201506994_sm_miscellaneous_information.pdf

Methods

Isolation and purification

The enzyme was isolated and purified as described previously.[1] Briefly, tyrosinase was extracted from frozen walnut leaves using phase separation steps based on detergent and PEG. The active form of the enzyme was then purified to homogeneity by applying two cation exchange chromatography steps (SP-Sepharose FF and MonoS HR 5/50 from GE) resulting in >90% purity according to SDS-PAGE. The purified enzyme was then concentrated to 10 mg ml^{-1} in 20 mM HEPES at pH 7.5 prior to crystallization.

Crystallization and structure determination

*jr*TYR crystals were grown applying the hanging drop vapour diffusion method after optimizing the hits from initial crystallization screening using the sitting drop vapour diffusion method.[2] 1 µl of 10 mg ml^{-1} *jr*TYR was mixed with 0.5 µl of reservoir solution (0.1 M MES pH 6.5, 30% PEG5000 MME (*w/v*), 0.2 M ammonium sulfate) and incubated at 293 K with single crystals appearing after a couple of days. Crystals were harvested with a nylon fibre loop (Hampton Research and MiTeGen) and cryo-protected (0.1 M MES pH 6.5, 30% PEG5000 MME (*w/v*), 0.2 M ammonium sulfate, 20% glycerol) before plunging them into liquid nitrogen. The enzyme crystallized in the space group C1 2 1 with unit cell dimensions a = 115.34 Å, b = 90.94 Å, c = 86.58 Å, α = 90.0°, β = 130.2° and γ = 90.0°. Diffraction data were collected on beamline P11 (λ = 1.033 Å) at DESY (Hamburg, Germany) using a PILATUS 6M detector. An oscillation range of 0.2 degree was chosen and 360° were collected. The obtained data were processed with XDS[3] and the structure was solved with programs from the CCP4[4] and PHENIX[5] suites by the means of molecular replacement (see Table S1). Initial phases were obtained by PHASER[6] using a homology model provided by BALBES,[7] which was fed with structure factors of *vv*CO (PDB entry: 2P3X, sequence identity: 36.08%) and the sequence of walnut tyrosinase.[1,8] The program AutoBuild[9] was then used for model building and the resulting model was subsequently refined with Phenix.refine. The final structure contains a single mutation (Ile instead of Val at position 107) and one of the two chains is missing six residues belonging to a large and very flexible loop (Ala157 - Gln194) due to low or no electron density. However, the structure shows excellent stereochemistry with 98% of the residues being Ramachandran favoured and no single outlier. The quality of the final model was evaluated by the MolProbity server and deposited in the Protein Data Bank with the PDB entry 5CE9.[10]

Enzyme kinetics

Kinetic data of walnut tyrosinase was measured using a spectrophotometric assay which detected the appearance of the product in the reaction solution. The *o*-quinones generated by the enzymatic action of tyrosinase were trapped by the potent nucleophile 3-methyl-2-benzothiazolinone hydrazone (MBTH) which couples to the generated *o*-quinones via its amino group generating adducts with high molar absorption coefficients that are reasonably stable.[11] Spectra were recorded on a Shimadzu UV-1800 spectrophotometer, the temperature in the cuvettes was maintained at 25 °C using a Julabo F25 MH thermostat in a circulating water-bath. Measurements were done in a final volume of 1 ml containing 50 mM sodium phosphate buffer pH 6.5, 5 mM MBTH, 2 % (*v/v*) of N,N-dimethylformamide as well as various concentrations of the substrate to be tested and *jr*TYR (1-125 nM).

Values for the molar absorption coefficients were determined from rapid oxidation of small concentrations of substrate under assay conditions but using enzyme concentrations in the µM range.

The parameters of the Michaelis-Menten model, K_m and v_{max}, were calculated from the steady-state rate of product formation for each of the initial substrate concentrations tested (with the exception of the two highest tested concentrations of tyramine and the highest concentration of *p*-tyrosol, respectively, which showed significant contributions of substrate inhibition.). All measurements were performed in triplicate; the reciprocals of the variances of the observed slopes were applied as weights for the nonlinear regression using the Levenberg-Marquardt algorithm[12] as implemented in the program Dataplot version 11/2010. Initial estimates for the two free parameters were obtained by applying the Hanes-Woolf linearization of the Michaelis-Menten equation.[13]

Figures and Tables

Table S1. Data collection and processing statistics for *jr*TYR

Space group	C 1 2 1
Wavelength (Å)	1.033
No. of images	1800
Oscillation (°)	0.2
Resolution range (Å)	44.1 - 1.8 (1.86-1.80)
Completeness (%)	99.7 (99.7)
R_{merge}[b]	0.098 (0.980)
$<I/\sigma(I)>$	12.03 (1.83)
Unit cell parameters (Å,°)	*a*=115.43, *b*=90.94, *c*=86.58, α=90 β=130.18, γ=90
$R_{p.i.m.}$[c]	0.041 (0.402)
$CC_{1/2}$	0.999 (0.855)
No. of reflections collected	429283
No of. Unique reflections	63119

[a] Values in parentheses are for the highest resolution shell. [b] $R_{merge} = \Sigma_{hkl} \Sigma_i |I_i(hkl)_i - <I(hkl)>|/\Sigma_{hkl} \Sigma_i I_i (hkl)_i$, where $I_i(hkl)$ is the ith observation of reflection hkl and $<I(hkl)>$ is the weighted average intensity for all observations of reflection hkl. [c] $R_{p.i.m.} = \Sigma_{hkl} \{1/[N(hkl) – 1]\}^{1/2} \Sigma_i|I_i(hkl) - <I(hkl)>|/\Sigma_{hkl} \Sigma_i I_i (hkl)$.

Table S2. Kinetic parameters for *jr*TYR

Substrate	λ_{max} / nm	ε_{max} / (M^{-1} cm^{-1})	K_m / mM	k_{cat} / s^{-1}
L-DOPA	**507**	**32900 ± 1600**	**41,3 ± 8,8[a]**	**1670 ± 350[a]**
L-Tyrosine	**507**	**32900 ± 1600**	**1,02 ± 0,036**	**2,70 ± 0,15**
Tyramine	**506**	**33300 ± 1100**	**0,274 ± 0,0018**	**18,3 ± 0,71**
p-Tyrosol	**499**	**36000 ± 1100**	**0,395 ± 0,0096**	**14,0 ± 0,54**

[a] Due to the solubility of L-DOPA which was too low for a reliable determination of K_m and k_{cat} (see Figure S2) the given numbers should be regarded as an indication of the order of magnitude rather than precise values for those two parameters. A pseudo first-order kinetic model assuming a substrate concentration negligibly small compared to K_m yields a value for k_{cat} / K_m of 40,9 mM^{-1} s^{-1} which compares favorably with the value of 40,4 mM^{-1} s^{-1} derived from the estimations given in the table.

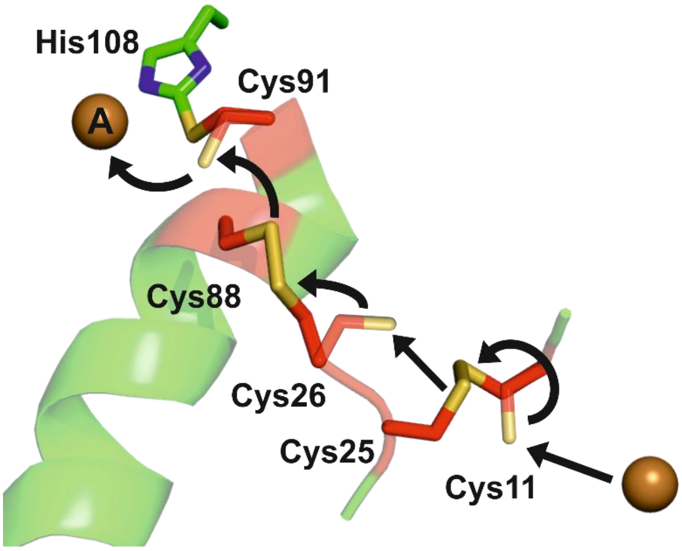

Figure S1. Copper incorporation scenario. The residues involved in the suggested copper incorporation scenario are shown as sticks. The carbon atoms of cysteine residues are colored in red and those of the CuA coordinating histidine residue are colored in green (color code: green/red = carbon, blue = nitrogen, yellow = sulfur). The remaining residues are shown as cartoon with 50% transparency, however, most of the surrounding structure was omitted for clearance. The disulfide bonds (Cys26-Cys88 and Cys11-Cys25) are shown in their oxidized form without transparency with single cysteine residues additionally illustrated in their reduced form (before the formation of the disulphide disulfide bond) with 30% transparency in order to better demonstrate the theoretical scenario of the suggested copper incorporation event. The arrows indicate the suggested pathway trajectory of the incoming copper, which is firstly bound by Cys11 (located on the protein surface) and then stepwisely transported into the active site via Cys25, Cys26, Cys88 and finally Cys91, which afterwards forms the thioether bridge with His108 autocatalytically. The remaining cysteines are then oxidized to form disulfide bonds. Note that this scenario is a theoretical suggestion.

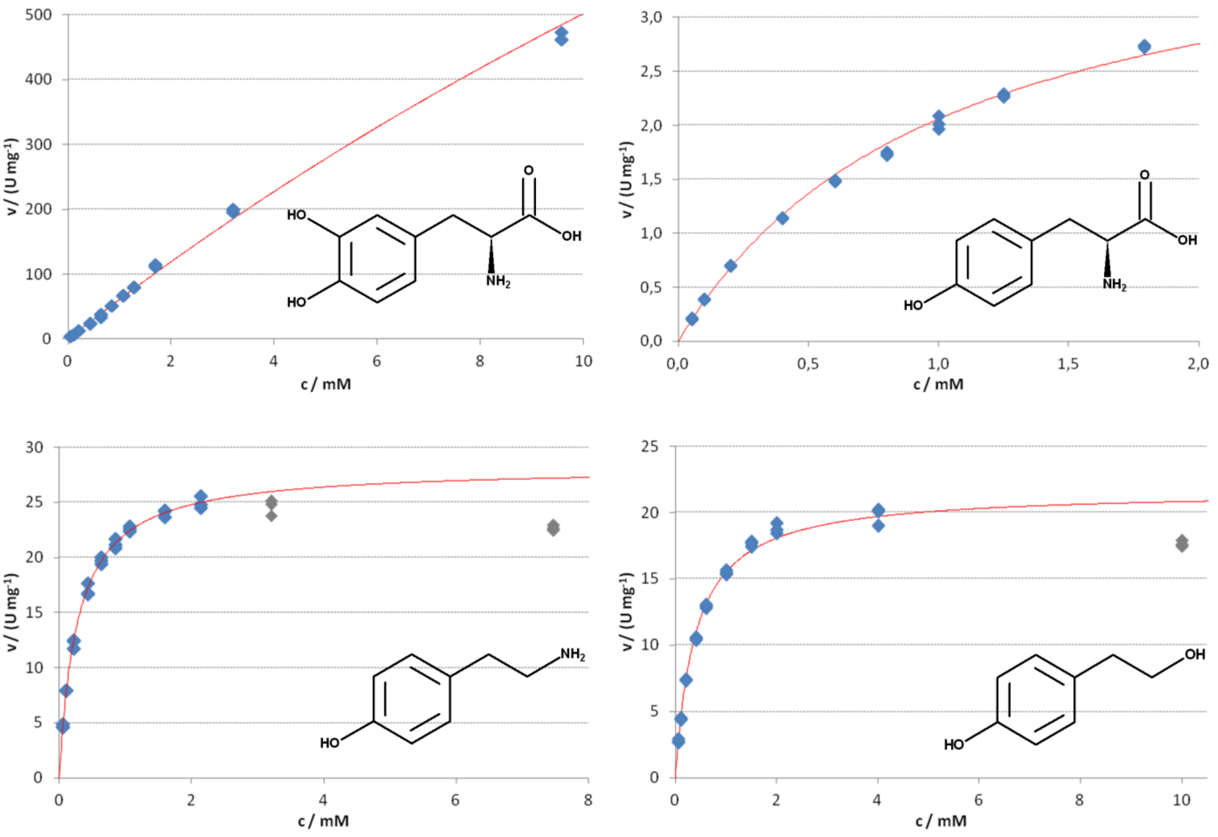

Figure S2. Reaction rate versus concentration for the substrates in table S2. Top left: *L*-DOPA, top right: *L*-tyrosine, bottom left: tyramine, bottom right: *p*-tyrosol; 1 U = 1 µmol min^{-1}; The blue diamonds represent measured slopes, grey diamonds represent measured slopes with significant contribution of substrate inhibition and the reaction rate predicted using the Michaelis-Menten model with the least-squares optimized parameters is shown as a red curve.

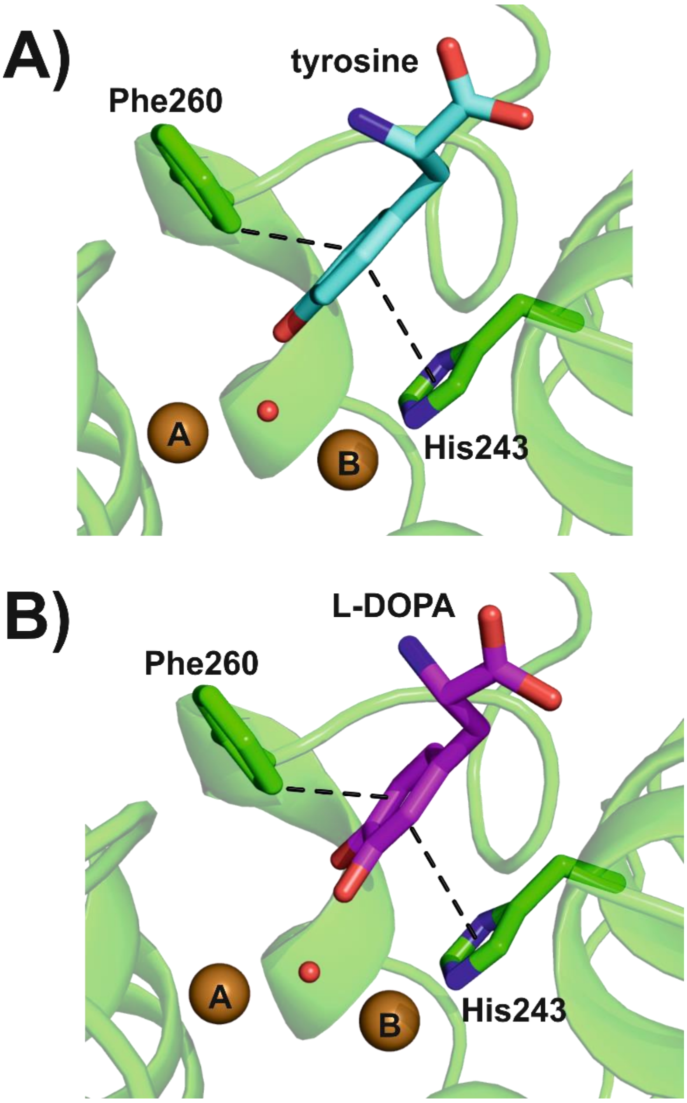

Figure S3. Gate formed by His243 and Phe260. A) Incoming tyrosine (from *bm*TYR + tyrosine structure, PDB entry: 4P6R)[14], illustrated in sticks representation (color code: cyan = carbon, blue = nitrogen, red = oxygen) is stabilized (shown by black dashes) by His243 and Phe260 (drawn as sticks, color code: green = carbon, blue = nitrogen). Both copper ions are represented as brown sphere with their bridging solvent molecule shown as small sphere, the rest of the structure is illustrated as cartoon with 50% transparency. B) L-DOPA (from *bm*TYR + tyrosine structure, PDB entry: 4P6R)[14], illustrated in sticks representation (color code: purple = carbon, blue = nitrogen, red = oxygen), is stabilized by the “gate residues” His243 and Phe260. The rest of the figure is shown as in A).

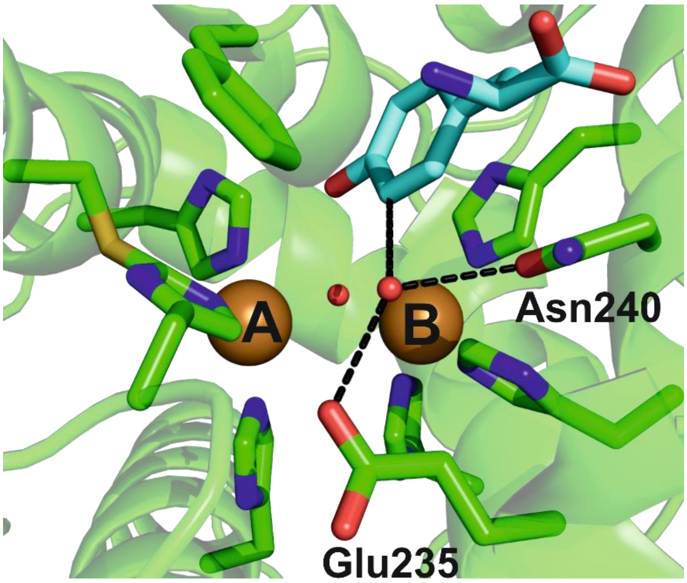

Figure S4. Substrate deprotonation. The conserved water molecule is shown as small red sphere with black dashes indicating its theoretical interactions. The water molecule is stabilized and activated by Glu235 and Asn240, which are shown as sticks (color code: green = carbon, blue = nitrogen, yellow = sulfur, red = oxygen). In addition a tyrosine (from *bm*TYR + tyrosine structure, PDB entry: 4P6R)[14] is illustrated in sticks representation (color code: cyan = carbon, blue = nitrogen, red = oxygen) in order to visualize the potential deprotonation interaction between the conserved water molecule and the *ortho*-position of the tyrosine. Moreover, both copper ions (brown spheres), the copper bridging solvent molecule (small red sphere), the six copper coordinating histidines and the blocker residue Phe260 (sticks, color code: green = carbon, blue = nitrogen, yellow = sulfur) are illustrated. Note that the illustrated *jr*TYR structure represents the *met*-form and that the deprotonation is assumed to take place with the enzyme being in its *oxy*-form (not illustrated).

References

[1] F. Zekiri, C. Molitor, S. G. Mauracher, C. Michael, R. L. Mayer, C. Gerner, A. Rompel, *Phytochemistry* **2014**, *101*, 5–15.

[2] F. Zekiri, A. Bijelic, C. Molitor, A. Rompel, *Acta Crystallogr. Sect. F Struct. Biol. Commun.* **2014**, *70*, 832–834.

[3] W. Kabsch, *Acta Crystallogr. D Biol. Crystallogr.* **2010**, *66*, 125–132.

[4] M. D. Winn, C. C. Ballard, K. D. Cowtan, E. J. Dodson, P. Emsley, P. R. Evans, R. M. Keegan, E. B. Krissinel, A. G. W. Leslie, A. McCoy, et al., *Acta Crystallogr. D Biol. Crystallogr.* **2011**, *67*, 235–242.

[5] P. D. Adams, P. V. Afonine, G. Bunkóczi, V. B. Chen, I. W. Davis, N. Echols, J. J. Headd, L.-W. Hung, G. J. Kapral, R. W. Grosse-Kunstleve, et al., *Acta Crystallogr. D Biol. Crystallogr.* **2010**, *66*, 213–221.

[6] A. J. McCoy, R. W. Grosse-Kunstleve, P. D. Adams, M. D. Winn, L. C. Storoni, R. J. Read, *J. Appl. Crystallogr.* **2007**, *40*, 658–674.

[7] F. Long, A. A. Vagin, P. Young, G. N. Murshudov, *Acta Crystallogr. D Biol. Crystallogr.* **2008**, *64*, 125–132.

[8] M. A. Escobar, A. Shilling, P. Higgins, S. L. Uratsu, A. M. Dandekar, *J. Am. Soc. Hortic. Sci.* **2008**, *133*, 852–858.

[9] T. C. Terwilliger, R. W. Grosse-Kunstleve, P. V. Afonine, N. W. Moriarty, P. H. Zwart, L.-W. Hung, R. J. Read, P. D. Adams, *Acta Crystallogr. D Biol. Crystallogr.* **2008**, *64*, 61–69.

[10] V. B. Chen, W. B. Arendall, J. J. Headd, D. A. Keedy, R. M. Immormino, G. J. Kapral, L. W. Murray, J. S. Richardson, D. C. Richardson, *Acta Crystallogr. D Biol. Crystallogr.* **2010**, *66*, 12–21.

[11] A. J. Winder, H. Harris, *Eur. J. Biochem.* **1991**, *198*, 317–326.

[12] D. Marquardt, *J. Soc. Ind. Appl. Math.* **1963**, *11*, 431–441.

[13] C. S. Hanes, *Biochem. J.* **1932**, *26*, 1406–1421.

[14] M. Goldfeder, M. Kanteev, S. Isaschar-Ovdat, N. Adir, A. Fishman, *Nat. Commun.* **2014**, *5*, 4505.
